# A New Approach for Mining Order-Preserving Submatrices Based on All Common Subsequences

**DOI:** 10.1155/2015/680434

**Published:** 2015-05-28

**Authors:** Yun Xue, Zhengling Liao, Meihang Li, Jie Luo, Qiuhua Kuang, Xiaohui Hu, Tiechen Li

**Affiliations:** Laboratory of Quantum Engineering and Quantum Materials, School of Physics and Telecommunication Engineering, South China Normal University, Guangzhou 510006, China

## Abstract

Order-preserving submatrices (OPSMs) have been applied in many fields, such as DNA microarray data analysis, automatic recommendation systems, and target marketing systems, as an important unsupervised learning model. Unfortunately, most existing methods are heuristic algorithms which are unable to reveal OPSMs entirely in NP-complete problem. In particular, deep OPSMs, corresponding to long patterns with few supporting sequences, incur explosive computational costs and are completely pruned by most popular methods. In this paper, we propose an exact method to discover all OPSMs based on frequent sequential pattern mining. First, an existing algorithm was adjusted to disclose all common subsequence (ACS) between every two row sequences, and therefore all deep OPSMs will not be missed. Then, an improved data structure for prefix tree was used to store and traverse ACS, and Apriori principle was employed to efficiently mine the frequent sequential pattern. Finally, experiments were implemented on gene and synthetic datasets. Results demonstrated the effectiveness and efficiency of this method.

## 1. Introduction

Recent numerous high-throughput developments in DNA chips generate massive gene expression results, which are represented as matrix *D* of real numbers with rows (objects) to represent the genes and columns (attributes) to represent the different environmental conditions, different organs, or even different individuals. Each element or entry represents the expression level of a gene under a specific condition.

To analyze the gene expression data, clustering is widely used to gather the objects into different clusters based on similarity. The objects in the same cluster are as similar as possible. Genes in the same cluster may show similar cellular function or expression mode, implying that they are more likely to be involved in the same cellular process. Similarity measurements are mainly based on distance functions, including the Euclidean distance and Manhattan distance. However, these distance functions are not appropriate to measure the object correlation in the gene matrix [1]. Moreover, only a small subset of genes participate in any cellular process of interest, and a cellular process occurs only in a subset of the samples, requiring biclustering or the subspace clustering to capture clusters formed by a subset of genes across a subset of samples [2].

**Figure 1:**
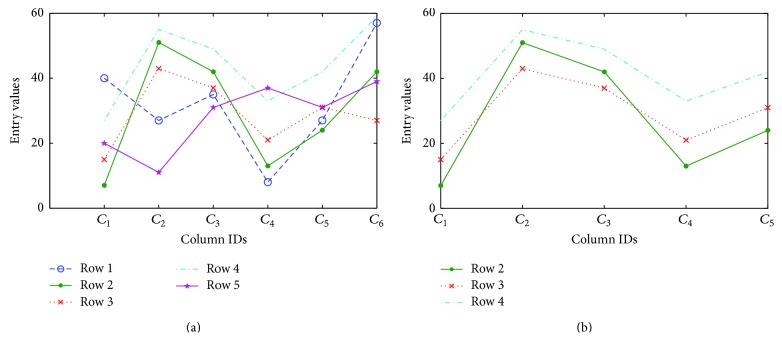
(a) Original data matrix: 5 rows and 6 columns; (b) three rows exhibit a coherent pattern.

Table 1 shows an example of the original 5 × 6 data matrix and the corresponding graph is shown in Figure 1(a). If all the rows or columns are considered, then the common mode could not be found. However, if the first five columns are considered, then the 2nd, 3rd, and 4th lines showed the same trend across these five columns as shown in Figure 1(b).

The problem is particularly true for gene expression analysis because the gene expression matrix usually has very high dimension [1]. However, the traditional clustering such as *K*-means [3] and hierarchical clustering [4] are difficult to use to identify these subsets. Given this observation with respect to the high dimensional data set, those embedded clusters attract wide concern in recent years [5–7], and many biclustering algorithms have been proposed to solve this problem [8–11]. Among them, the pattern-based subspace clustering, which is based on the pattern similarity rather than the distance similarity, has been widely applied in the analysis of gene expression, recommender systems, target sales, and so on.

The typical microarray data sometimes has high level noise. Coregulation genes do not necessarily have the same absolute expression level. So to make a comparison of different genes in different experiments, the relative expression levels are more meaningful than their absolute values. Interesting biological knowledge is usually concealed in the genes, which show a similar pattern (rises and falls) in different experimental conditions.

**Figure 2:**
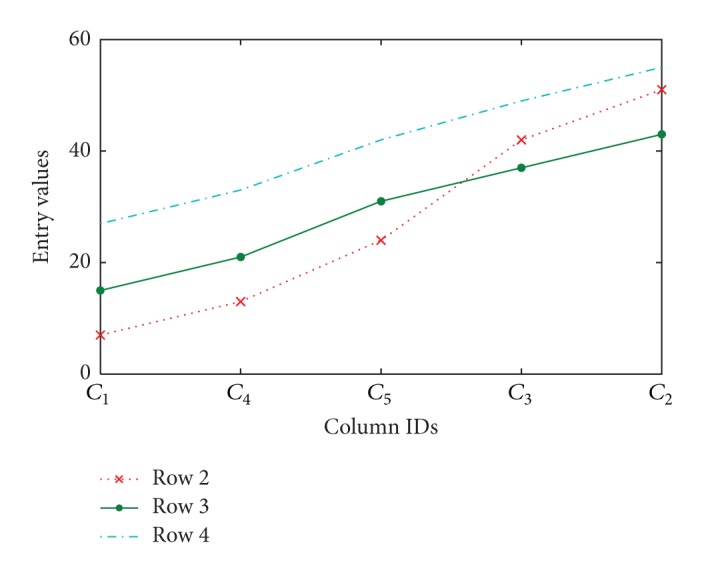
Three rows form a coherent ascending pattern under permutated columns.

This paper focuses on pattern-based subspace clustering, also known as order-preserving submatrix (OPSM) model. A noncontiguous submatrix is OPSM provided column permutation exists, such that the values in all the rows of the submatrix are strictly monotonically increasing. The tendency among the elements matters more to the model than the actual values.  Figure 2 shows that the sequences are monotonically increasing under the new column order given that columns are rearranged. In the field of biology, OPSM model has been accepted as a biologically meaningful cluster model. In addition, the model can also be used in business forecasting. For example, the customers are divided into several categories according to the customer scoring on the telecom tariff packages. Customers who belong to the same class have the same needs such as internet connectivity and surfing speed. The market manager can devise different market strategies for different customer groups based on the results.

If each row vector is sorted in an ascending order with the column indices replacing the original value, then the original matrix is transformed into data set of sequences and OPSM mining problem is simplified as a special case of frequent sequential pattern mining [12]. A frequent sequential pattern is uniquely defined as OPSM with all the supporting sequences as rows. The length of a sequential pattern is the number of columns included. Supporting count is the number of rows containing the sequence. A sequential pattern whose supporting count is beyond a minimum support threshold, min sup, is also known as frequent sequential pattern. Therefore, the problem of mining significant OPSM is equivalent to the search for the complete set of frequent sequential patterns.

Most existing sequential pattern mining methods rely on setting minimum support threshold to narrow the search space. Given that the small support threshold will cause the explosive growth of the calculation cost, most of the existing methods improve the efficiency of the algorithm by setting a comparatively larger threshold. However, the large supporting threshold could not find the deep OPSM. The concept of deep OPSM with long patterns and small supporting row count was first proposed by Gao et al. [12]. Deep OPSMs are significant to biologists because they may represent small groups of genes that are tightly coregulated under some conditions. In some important biological processes, such as protein-protein interactions, and biological pathway membership, only a limited number of genes are involved in these processes. However, the general algorithms for frequent sequential pattern mining usually ignore this type of OPSMs.

To solve the above problems, this paper transforms OPSM into frequent sequential pattern first, and then an exact algorithm is proposed to search OPSMs based on frequent common subsequence mining. It can mine all OPSMs embedded in a given matrix and provide flexibility for row and column supports, which allows the discovery of deep OPSMs.

An algorithm calACS proposed by Wang and Lin [13] was improved to determine all common subsequences between two sequences. Then, the Apriori rules were introduced to narrow the search space, and the prefix tree was constructed to store and traverse the sequence modes to reduce time and space complexity. Finally, all OPSMs satisfied the defined threshold. In our algorithm, the computation cost would not increase enormously even if the value of the threshold was very small.

The rest of this paper is organized as follows. In Section 2, we review some related works.  Section 3 defines OPSM.  Section 4 describes the algorithm and the data structure.  Section 5 reports the experimental results.  Section 6 concludes the paper.

## 2. Related Work

Subspace clustering determines the embedded clusters in high dimensional data set. Hartigan [14] first proposed to cluster rows and columns simultaneously. Cheng and Church [15] applied it for knowledge discovery in the expression of gene data. The method overcame the weakness of traditional clustering methods, allowing for the simultaneous clustering of genes and conditions. If the mean square error of submatrix *A* is less than *δ*, then submatrix *A* is a bicluster. A greedy algorithm is proposed to search submatrices with low mean square error in a gene expression matrix, which are consistent biclusters. These submatrices performed well to determine coregulation patterns in genes and attributes [15].

Ben-Dor et al. [16] first proposed OPSM mining model, which pertained to the relative value of entry rather than the actual value. OPSM is essentially a pattern-based subspace clustering. The subset of a matrix is OPSM when the value of each row is strictly increasing or decreasing under column permutation. They proved that the problem is NP-hard and presented a greedy heuristic algorithm for mining OPSM. The algorithm can mine some OPSMs with large row support but cannot guarantee that all OPSMs could be found.

Cheung et al. [1] proposed a maximal OPSM model, converting OPSM problem into a sequential pattern mining problem. To mine all maximal OPSMs with a candidate generation-and-test framework, a new data structure head-tail tree was introduced. However, their algorithm is based on the Apriori principle, and thus the number of maximal OPSMs was affected by supporting row threshold, which increases in proportion with database size.

Gao et al. [12] proposed a new model also known as deep OPSM, referring to long patterns with a few supporting sequences. Deep OPSMs have biological significance. A framework KiWi was proposed to mine deep OPSMs in massive data sets effectively. Two parameters *k* and *w* were exploited to bound existing computing resources and determine as many deep OPSMs as possible. However, the algorithm was heuristic, which cannot guarantee the finding of all deep OPSMs.

## 3. OPSM Problem

In this section, we defined OPSM and detailed the process of transforming OPSM into the problem of mining frequent common subsequences.

Consider *n* × *m* data matrix *D*, where *R* is the row set and *C* is the column set in *D*. *d*_*ij*_ is the entry whose row label is *i* and column label is *j*. A cluster *S* = (*R*_*S*_, *C*_*S*_) is a submatrix of *D*, where *R*_*S*_ is a subset of *n* rows and *C*_*S*_ is a subset of *m* columns. The rows and columns do not need to be contiguous in *D*.

**Definition 1 .**

Submatrix *S* is OPSM if there exists a permutation of *C*_*S*_. The entries of each row in *R*_*S*_ are strictly monotonically increasing. For example, Table 1 displays a 5 × 6 matrix. If rows 2, 3, and 4 are increasing from *C*_1_ to *C*_2_, then ({2,3, 4}, 〈1,2〉) is OPSM. The fundamental goal is to find all the significant OPSMs in a given data matrix *D*.

In the data preprocess, each row is sorted in an ascending order, and the values are replaced by the original column label. Then, the original matrix is transformed into data set of sequences. The original data matrix of Table 1 is modified into the data set of sequences shown in Table 2. If the values of two entries in a row are the same, then the one that appears earlier is placed in front. A sequential pattern is frequent when the support of the sequence is greater than a predefined minimum support threshold, min sup. Therefore, OPSM mining problem can be simplified as a special case of frequent sequential pattern mining. A frequent sequential pattern uniquely defines OPSM, in which the sequential pattern is composed of OPSM columns, and the support sequence comprises the rows of OPSM.

Most existing sequential pattern mining methods search OPSMs by finding all the sequences whose support is greater than a given minimum support threshold. The efficiency of the mining algorithm is very sensitive to the minimum support threshold. A larger threshold is adopted to narrow the search space and reduce the complexity of the algorithm because a small threshold results in the high cost of computation. However, this method ignores some statistically and biologically significant OPSMs, deep OPSMs. Deep OPSMs are OPSMs with comparatively more columns and fewer rows that cannot be efficiently discovered by traditional methods [12].

To solve this problem, a new exact algorithm is proposed in this paper. The first step is to determine all common sequences from each two rows in the data set to form the candidate patterns with arbitrary length whose support is at least 2. Then, the database is scanned to calculate the row support for the candidate patterns whose length is 2 to find all the frequent sequential patterns with length 2. The third step is to construct the prefix tree and store the frequent sequential patterns (with length 2). The fourth step is to traverse the prefix tree and insert the node in the branch based on the Apriori principle and calculate the support again to obtain the frequent sequential patterns whose length is 3. The algorithm runs iteratively until all OPSMs satisfying the minimum support threshold could be found. In this process, if larger support threshold is not used to prune, then the results will contain all the deep OPSMs.

## 4. Algorithm

### 4.1. All Common Subsequences

All common subsequence (ACS) [17] is a variation from the traditional longest common subsequence (LCS). LCS is a classical problem with a goal to determine LCS from a set of sequences (generally two sequences). Wang [17] proposed this new method to calculate the similarity between two sequences. Different from the previous LCS method, this method calculates the similarity based on the number of all common sequences between the two sequences. calACS [13] is a new method to calculate the number of ACS between sequences *A* and *B*. We improved calACS to obtain all common subsequences between two sequences. The pseudocode of the improved calACS algorithm is shown as Algorithm 1.

As shown in the pseudocode, *N*_*A*_[*i*] stores the common subsequences whose end is element *A*_*i*_ [line 6]. Provided that any two items in the common sequence remained in the same order in sequences *A* and *B*, for any *j* < *i*, if item *A*_*j*_ in *B* sequence is arranged before item *A*_*i*_, the same order in sequence *A* is retained [line 17]. Hence, the common subsequence ending with *A*_*i*_ must contain the common subsequence ending with *A*_*j*_. They are combined to form the new common sequences and all common subsequence of *A* and *B* is the union of *N*_*A*_[*i*] [line 18].

We use a prefix tree to store and traverse all common sequences. Different from the traditional method to solve OPSM problem, frequent common subsequences can be obtained by traversing frequent prefix tree rather than by the columns joint.

The prefix tree, also known as* trie*, is an ordered tree used to store strings or associative arrays, in which the nodes from the root to the leaf form a path. The root node is null corresponding to an empty sequence. The common nodes store the column indices and the leaf nodes retain the row indices, which support the branch (a branch is a common subsequence). The sequence is composed of *K* nodes known as *K* sequence as shown in Figure 4. A right path (5,4) in the tree and the leaf node preserves the number (3,4, 5). That is, rows 3, 4, and 5 have common subsequence (5,4).

Suppose a complete set of ACS is obtained, such as *S* = (*R*_*ij*_, 〈*C*_1_, *C*_2_,…, *C*_*k*_〉). *R*_*ij*_ represents the labels of rows *i* and *j*. *C*_*i*_ is the element and *k* is the length of the common subsequence, indicating that 〈*C*_1_, *C*_2_,…, *C*_*k*_〉 is ordered. We insert sequence *S* into the prefix tree whose path is 〈*C*_1_, *C*_2_,…, *C*_*k*_〉 and record *R*_*ij*_ in leaf nodes, which support the sequence.

The traditional method to construct a prefix tree is described in the subsequent paragraphs.

First, we traverse the prefix tree by preorder. If the first *k* prefix of length *K* + 1 sequence is the same as length *K* path in the prefix tree, then (*K* + 1)th node will be added to the path tail before the leaf node. As the length *K* + 1 sequence is different from length *K* sequence, the corresponding leaf node will be revised, and the rows will be recounted to obtain the support of length *K* + 1 path.

However, if data sets are high dimensional and very dense [12], then the prefix tree will become enormous and occupy a huge space when new sequences are added. Traversing and intersection operations are also time-consuming. Hence, reducing the computational complexity is necessary. In this paper, we develop two kinds of prefix trees, namely, candidate and frequent trees, to save the candidate and frequent sequential patterns, and use the Apriori principle to narrow the search space of patterns.

According to the Apriori principle, if a length *K* sequence is frequent, then all of its subsequences must be frequent; in other words, if a length *K* sequence has length *K* − 1 subsequence which is not frequent, then length *K* sequence must not be frequent either. Thus, if length *K* − 1 subsequence which is formed by the first *K* − 1 items of length *K* sequence is not a branch in the frequent tree, the length *K* sequence should not be inserted into the candidate tree.

In Section 4.1, calACS is introduced to obtain ACS between any two sequences. The common subsequences with length 2 are employed to generate the 2-candidate prefix tree. The 2-candidate tree is constructed via traversing and inserting with each path retaining the column indices, and all the leaf nodes store the corresponding support row indices, as well as the number of the support rows. Furthermore, we use the number of the support rows to determine whether a branch (i.e., a path) is frequent. If the branch satisfies the support threshold (min sup), it is preserved; otherwise, it is pruned. After all the prune operation is performed, the 2-frequent tree is obtained, which is the first iteration.

The next step is to add the common subsequences with length 3 to the 2-frequent tree. The process is as follows.

Preorder traverses the 2-frequent tree. If the first two prefixes of the length 3 common subsequence are the same as a branch in 2-frequent tree, then the third node is added to the tail of the branch and the leaf node is simultaneously updated, restoring the support row indices and recounting the number of rows.

By contrast, if the first two prefixes of the length 3 common subsequence do not match any path in 2-frequent tree, according to the Apriori principle, then the length 3 common subsequence must not be frequent either and should not be added as a path to the prefix tree. This process reduced the unnecessary traversal and the comparison between sequences, which are very time-consuming in a large prefix tree. Thereafter, we obtain the 3-candidate tree. After pruning infrequent branches, the 3-frequent tree is acquired.

The above process is repeated to generate *K*-candidate tree from *K* − 1 frequent tree. Prune the branches which do not meet the minimum support threshold to obtain *K*-frequent tree, in which each path or branch is a frequent sequence. The program is not terminated until the common subsequences with the longest length are visited. The final result is a tree with the longest path to satisfy the support. The nodes in each path represent the column indices, and the leaf node of each path stores the corresponding row indices. Thus, all OPSMs can be found.

**Figure 3:**
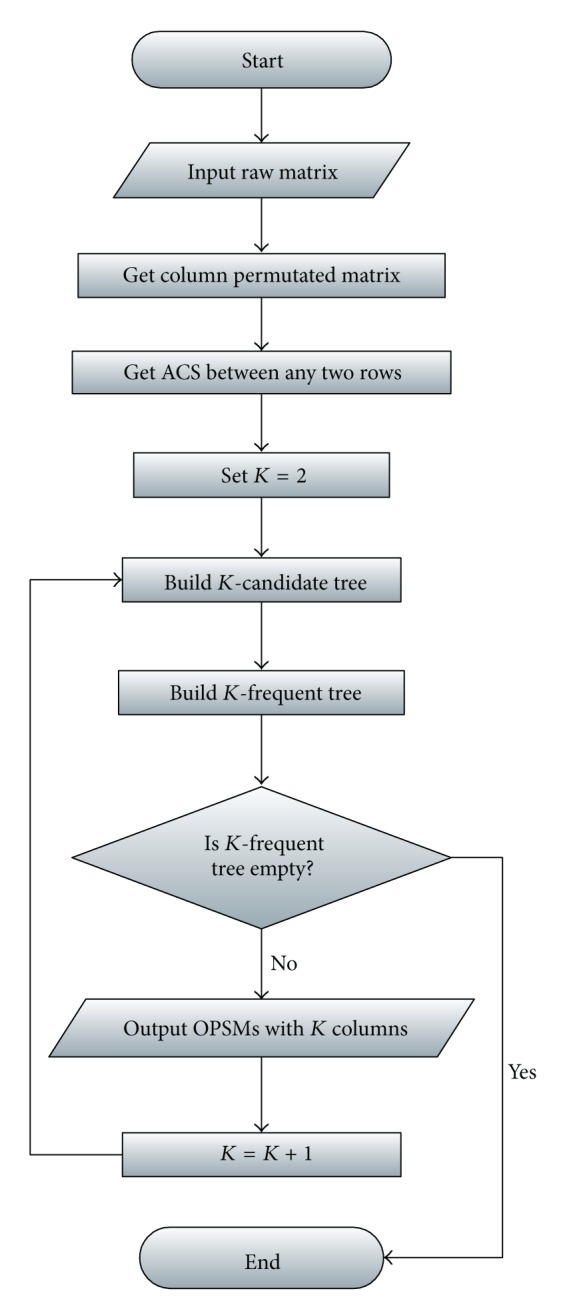
Flowchart of our algorithm.

The flowchart of our algorithm is as Figure 3.

### 4.2. An Example to Find ACS

Given an original *M* × *N* data matrix *D*, where *d*_*ij*_ represents the expression level of the gene *i* under the condition *j*, a matrix is shown in Table 3. When each row in the matrix *D* is sorted in an ascending order and their values are replaced by the corresponding column indices, the matrix is replaced with a new matrix *C* as shown in Table 4. ACS could be obtained by applying the improved calACS algorithm for matrix *C*.

Common subsequences from arbitrary two rows are shown in Table 5. However, the relatively large space complexity results in great inconvenience for later traversal, storage, and support calculations. Hence, a prefix tree is adopted for faster operation to reduce the space complexity.

### 4.3. Construct **ζ**-Frequent Prefix Tree

**Figure 4:**
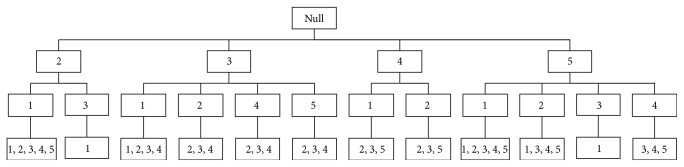
Example of two-candidate prefix tree.

Firstly, *ζ*-candidate prefix tree would be generated by the candidate *ζ*-subsequences matrix. Figure 4 illustrates the 2-candidate prefix tree for *ζ* = 2 after finding ACS operation. The leaf nodes of the prefix tree store the labels of the rows of a common subsequence (a branch). For example, the leaf node of the right branch in Figure 4 records (3,4, 5), implying that rows 3, 4, and 5 have common subsequence whose column heads are 5 and 4.

The sequences in the leaf nodes of *ζ*-candidate prefix tree do not necessarily have to be frequent because the *ζ*-candidate prefix tree would be used to generate the *ζ*-frequent prefix tree whose leaf nodes are frequent subsequences with length *k*.

**Figure 5:**
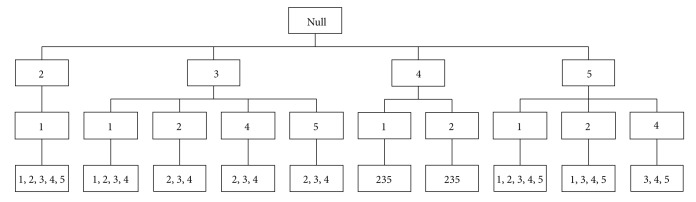
Example of 2-frequent prefix tree with “*δ* = 3.”

*ζ*-frequent prefix tree is constructed by deleting the infrequent subsequences that dissatisfy the minimum support *δ*.  Figure 5 is an example of *ζ*-frequent prefix tree (*ζ* = 2).

### 4.4. Build the (**ζ** + 1)-Frequent Prefix Tree

Specific steps are detailed to construct (*ζ* + 1)-candidate prefix tree. Based on Apriori principle, if a sequence is frequent, then all of its subsequences must be frequent. Only the frequent sequences can generate the supersequence.

**Figure 6:**
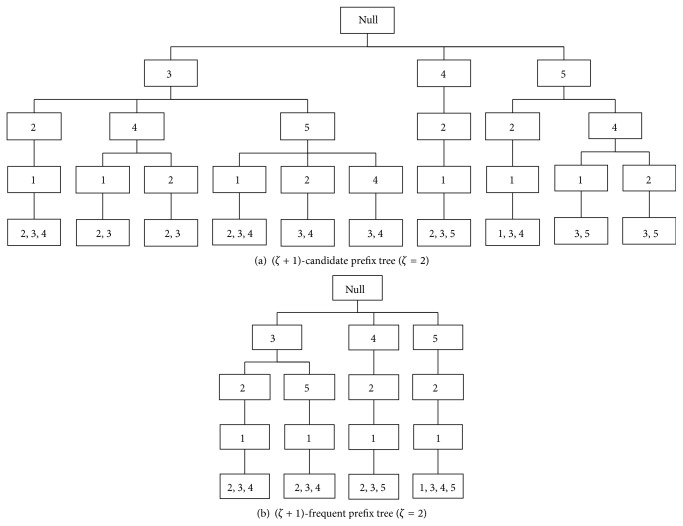
Results of (*ζ* + 1)-frequent prefix tree mining.

To build (*ζ* + 1)-frequent prefix tree, first the (*ζ* + 1)th element of the common (*ζ* + 1) subsequences is inserted into (*ζ* + 2)th layer of *ζ*-frequent prefix tree. At the same time, the leaf nodes of *ζ*-frequent prefix tree are revised. Second, the infrequent subsequences of (*ζ* + 1)-candidate prefix tree are rejected, and (*ζ* + 1)-frequent prefix tree is established in this way. Moreover, (*ζ* + 1)-candidate prefix tree and (*ζ* + 1)-frequent prefix tree of the example are shown in Figure 6.

## 5. Experiments and Results

### 5.1. The Experiment on Gene Data Set

The algorithm was implemented on the platform of MATLAB R2011b with i3 380CPU and 4G memory, and the operating system was Windows Server 2007. The real data set was yeast galactose data of [18, 19], which was 205 × 80 real microarray data set obtained from a study of gene response to the knockout of various genes in galactose utilization (GAL) pathway of baker's yeast, with columns corresponding to the knockout conditions and rows corresponding to genes that exhibit responses to the knockouts. The experimental data set is 160 × 40 microarray data set by deleting 45 contiguous rows and 40 columns from the original matrix.

#### 5.1.1. Overlap

BicAT software and MATLAB were used in our experiments [20], and overlap is defined as follows [21].

Let *G*_1_, *G*_2_ be two gene sets in biclusters. The overlap of *G*_1_ and *G*_2_ is their intersection divided by their union, and 1 means module identity and 0 means no overlap. Consider

(1) $$\begin{array}{c} S_{G}\left(G_{1},G_{2}\right)=\frac{\left|G_{1}\cap G_{2}\right|}{\left|G_{1}\cup G_{2}\right|}. \end{array}$$

The experimental results are filtered in two steps.

1. If a bicluster contains another, then the smaller bicluster will be abandoned.
2. The column threshold is set. For example, if the threshold is six, then the biclusters whose column numbers are less than six will be discarded.

Finally, we obtained all the biclusters corresponding to column threshold six. The total number of OPSMs obtained is shown in Table 6. We can mine all OPSMs that meet the row threshold because our algorithm is exact. The number of OPSMs decreases as the number of row threshold increases.

**Figure 7:**
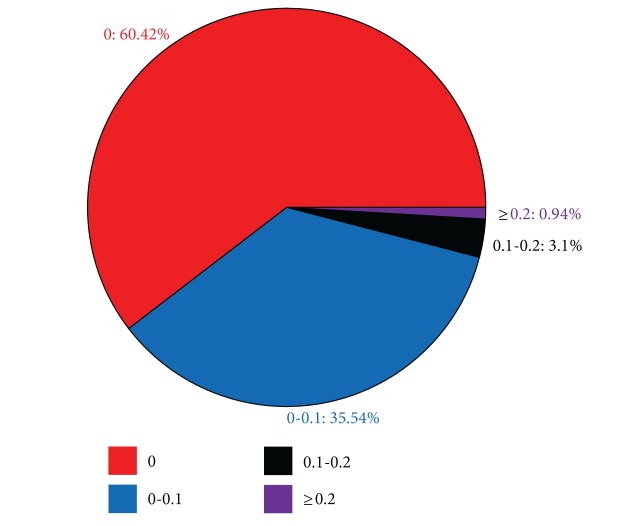
Statistical chart of the overlap distribution.

Furthermore, Figure 7 shows the statistical chart on the overlap distribution of 771 biclusters whose row threshold is 10 and column threshold is 6.

Figure 7 shows that no-overlap biclusters accounted for 60.42% of the total, and the degree of overlap between 0 and 0.1 (excluding 0) accounted for 35.54% of the total. Therefore, the biclusters whose overlap was between 0 and 0.1 (including 0) accounted for 95.96%. That is, the biclusters have no overlap or very small overlap.

#### 5.1.2. An Example of Mined OPSMs

**Figure 8:**
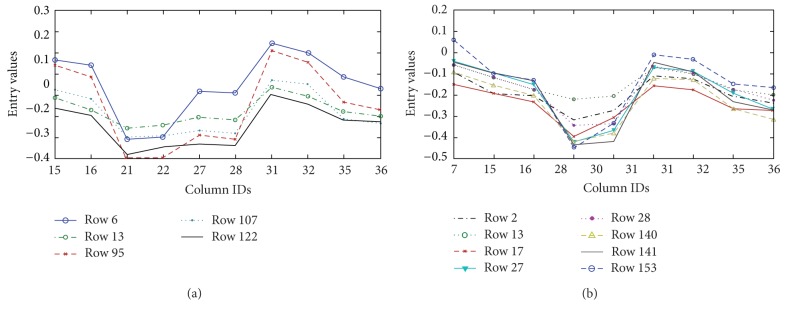
Two examples of mined OPSMs on gene data set.

Figures 8(a) and 8(b) show OPSMs that contain the maximal number of columns when the row threshold was set to five and eight.  Figure 8(a) shows five genes whose expression values exhibit simultaneous rise and fall across 10 different experiments.  Figure 8(b) shows the maximum number of columns that identify OPSM when the row threshold is eight.

#### 5.1.3. Enrichment

**Figure 9:**
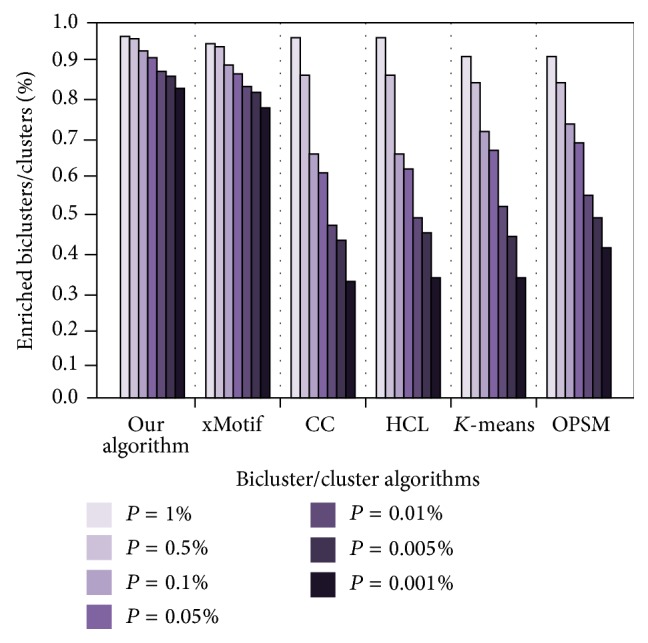
Percentage of significant enriched biclusters/clusters by GO Biological Process category for the five selected biclustering methods and our algorithm at different significance levels *P*.

The experimental data set is 160 × 40 yeast data set. We first use CC, HCl, *K*-means, OPSM, and xMotif model in the BicAT toolbox to obtain the results. Then, we run our program to obtain the corresponding result. The results obtained are packaged, respectively, in GO analysis tool (http://go.princeton.edu/cgi-bin/GOTermFinder) to obtain their *P* values. Finally, all the results are sorted and analyzed.  Figure 9 compares the enrichment results [22, 23].

Figure 9 shows that the enrichment of our algorithm is significantly higher than the enrichment of CC, HCl, *K*-means, and OPSM. In particular, the smaller *P* value can show our advantage. The results of xMotif algorithm were close to ours, but slightly less.

### 5.2. Experiments on Synthetic Data Set

#### 5.2.1. The Influence of Noise

The generation of the simulated data is as follows. First, we generated 30 × 15 standard normal distribution matrix as the initial matrix with five embedded nonoverlapping 5 × 3 OPSMs whose row and column sets were recorded. Then, we generated different levels of noise whose means were 0 and variances were 0, 0.002, 0.004, 0.006, 0.008, and 0.01, respectively. The noise will be added to the initial matrix. Finally, we obtained six input matrices with different noise levels.

We introduced match score to evaluate the algorithm [22]. Let *M*_1_ and *M*_2_ be two bicluster sets. Then, the gene match score of *M*_1_ with respect to *M*_2_ is defined as

(2) $$\begin{array}{c} S_{G}^{*}\left(M_{1},M_{2}\right)=\frac{\sum_{\left(G_{1},C_{1}\right)\in M_{1}}^{}max_{\left(G_{2},C_{2}\right)\in M_{2}}S_{G}\left(G_{1},G_{2}\right)}{\left|M_{1}\right|}. \end{array}$$

It shows the average of the maximum gene match scores for all biclusters in *M*_1_ with respect to the biclusters in *M*_2_. An overall match score can be interpreted as $S^{*}(M_{1},M_{2})=\sqrt{S_{G}^{*}\left(M_{1},M_{2}\right)\cdot S_{C}^{*}(M_{1},M_{2})}$, where *S*_*C*_^*∗*^(*M*_1_, *M*_2_) is the corresponding condition match score.

**Figure 10:**
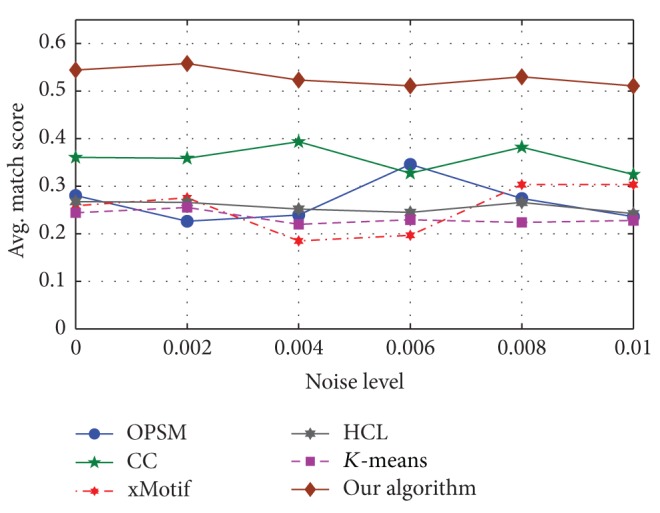
Effect of noise.

We calculated the match score of different bicluster results, and the comparison was as shown in Figure 10 [22].

The match score of our algorithm is better than others. As the level of noise increases, the total match score decreases slowly.

#### 5.2.2. Overlap

**Figure 11:**
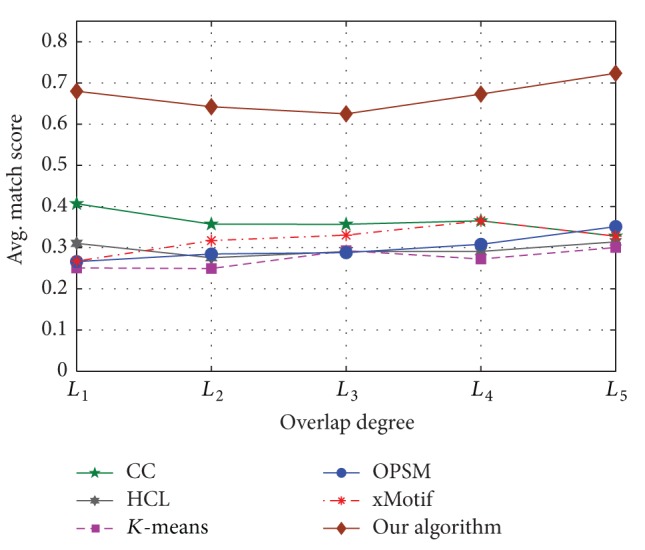
Effect of overlap.

First, we generated 30 × 15 standard normal distribution matrix with five embedded 5 × 4 OPSMs whose row and column sets were recorded. Similarly, we obtained five input matrices with different overlap levels. The levels of overlap were *L*_1_, *L*_2_, *L*_3_, *L*_4_, and *L*_5_ corresponding to 0, 0.087, 0.1905, 0.3158, and 0.4706, respectively. The synthetic data were tested by different algorithms, and the match score of all the results was calculated. The performance comparison is shown as in Figure 11 [22].

Figure 11 shows that the match score of our algorithm was better than other algorithms. The parameter settings of other algorithms were based on the best experimental results.

## 6. Conclusion

OPSMs have been accepted as a biologically meaningful bicluster model. Deep OPSMs consisting of a small number of genes sharing expression patterns over many conditions are very interesting to biologists.

In this paper, an exact algorithm was proposed based on frequent sequential patterns to mine not only all OPSMs, but also the deep OPSMs. The experiment on the gene data set showed that this approach can discover the biological significant OPSMs and deep OPSMs exhaustively. Moreover, the experimental results for synthetic data sets proved that our method can effectively mine the implanted biclusters under different noise and overlap levels.

## Figures and Tables

**Algorithm 1:**
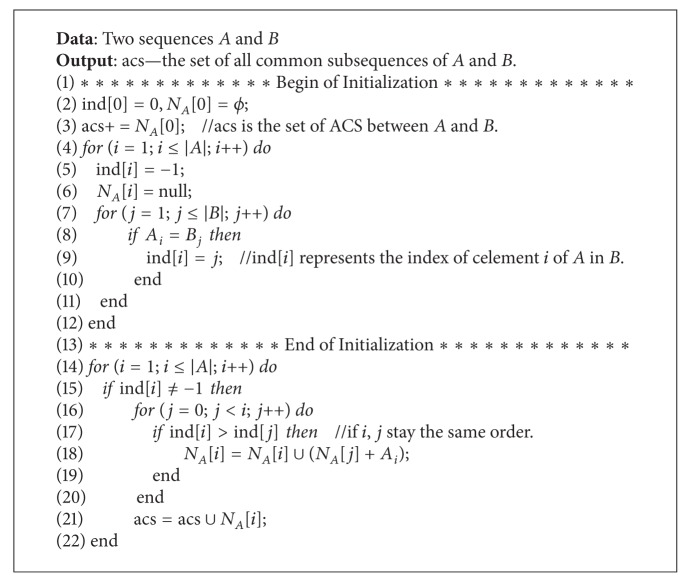


**Table 1:** Raw data matrix.

Rows	Columns					
	1	2	3	4	5	6
Row 1	40	27	35	8	27	57
Row 2	7	51	42	13	24	42
Row 3	15	43	37	21	31	27
Row 4	27	55	49	33	42	59
Row 5	20	11	31	37	31	39

**Table 2:** Transformed sequence data sets.

Rows	Columns					
	1	2	3	4	5	6
Row 1	4	2	5	3	1	6
Row 2	1	4	5	3	6	2
Row 3	1	4	6	5	3	2
Row 4	1	4	5	3	2	6
Row 5	2	1	3	5	4	6

**Table 3:** A microarray data matrix *D*.

Rows	Columns				
	1	2	3	4	5
Row 1	120	110	119		100
Row 2	999	128	80	115	810
Row 3	676	300	77	287	264
Row 4	197	107	99	587	101
Row 5	154	78		20	10

**Table 4:** An example of column permutated matrix *C*.

Rows	Columns				
	1	2	3	4	5
Row 1	5	2	3	1	
Row 2	3	4	2	5	1
Row 3	3	5	4	2	1
Row 4	3	5	2	1	4
Row 5	5	4	2	1	

**Table:** (a) Candidate 2-subsequences matrix

Sequences	Common 2-subsequences

1, 2	5, 1; 2, 1; 3, 1
1, 3	5, 2; 5, 1; 2, 1; 3, 1
1, 4	5, 2; 5, 1; 2, 1; 3, 1
1, 5	5, 2; 5, 1; 2, 1
2, 3	3, 4; 3, 2; 3, 5; 3, 1; 4, 1; 4, 2; 2, 1; 5, 1
2, 4	3, 4; 3, 2; 3, 5; 3, 1; 2, 1; 5, 1
2, 5	4, 2; 4, 1; 2, 1
3, 4	3, 5; 3, 4; 3, 2; 3, 1; 5, 4; 5, 2; 5, 1; 2, 1
3, 5	5, 4; 5, 2; 5, 1; 4, 2; 4, 1; 2, 1
4, 5	5, 2; 5, 1; 5, 4; 2, 1

**Table:** (b) Candidate 3-subsequences matrix

Sequences	Common 3-subsequences

1, 3	5, 2, 1
1, 4	5, 2, 1
1, 5	5, 2, 1
2, 3	3, 4, 2; 3, 4, 1; 3, 2, 1; 3, 5, 1; 4, 2, 1
2, 4	3, 5, 1; 3, 2, 1
2, 5	4, 2, 1
3, 4	3, 2, 1; 3, 5, 4; 3, 5, 1; 3, 5, 2; 5, 2, 1
3, 5	5, 4, 2; 5, 4, 1; 5, 2, 1
4, 5	5, 2, 1

**Table:** (c) Candidate 4-subsequences matrix

Sequences	Common 4-subsequences

2, 3	3, 4, 2, 1
3, 4	3, 5, 2, 1

**Table 6:** Number of OPSMs of different row thresholds.

The row threshold	3	5	8	10

Number of biclusters	9248	2791	1350	771
